# Comparing Metabolites and Functional Properties of Various Tomatoes Using Mass Spectrometry-Based Metabolomics Approach

**DOI:** 10.3389/fnut.2021.659646

**Published:** 2021-04-08

**Authors:** Ha In Mun, Min Cheol Kwon, Na-Rae Lee, Su Young Son, Da Hye Song, Choong Hwan Lee

**Affiliations:** ^1^Department of Bioscience and Biotechnology, Konkuk University, Seoul, South Korea; ^2^Research Institute for Bioactive-Metabolome Network, Konkuk University, Seoul, South Korea

**Keywords:** tomato, MS-based metabolomics approach, metabolic pathway analysis, functional properties, physicochemical properties

## Abstract

Tomato is one of the world's most consumed vegetables, and thus, various cultivars have been developed. Therefore, metabolic differences and nutrient contents of various tomatoes need to be discovered. To do so, we performed metabolite profiling along with evaluation of morphological and physicochemical properties of five representative tomato types. Common tomato cultivars, bigger and heavier than other tomatoes, contained higher levels of amino acids, organic acids, and lipids. On the contrary, cherry tomato cultivars contained a higher proportion of phenylpropanoids, lycopene, β-carotene, and α-carotene than the other tomatoes. Also, the highest antioxidant activity and total phenolic and flavonoid contents were observed in cherry tomato cultivars. Furthermore, to understand metabolic distributions in various tomato cultivars, we constructed a metabolic pathway map. The higher metabolic flux distribution of most primary metabolite synthetic pathways was observed in common tomatoes, while cherry tomato cultivars showed a significantly elevated flux in secondary metabolite synthetic pathways. Accordingly, these results provide valuable information of different characteristics in various tomatoes, which can be considered while purchasing and improving tomato cultivars.

## Introduction

Tomato (*Lycopersicon esculentum* Mill.) is one of the superfoods and known as an intensely nutritious and healthy food (1). Also, the health beneficial effects of tomatoes such as antioxidant, anti-inflammatory, anti-cancer, and anti-atherogenic properties, reducing risk of cardiovascular diseases, have been intensively studied (2–5). Due to the benefits of tomatoes and growing interest in health, it became a highly popular fruit (6). The worldwide production of tomato has been gradually increased from 153.31 Mt (in 2010) to 182.26 Mt (in 2018) (7). In order to follow food market trends, a myriad of new tomatoes, over 10,000 cultivars, have been developed to improve flavors, nutrients, and appearances (8, 9). Although many kinds of tomatoes provided more options to consumers, they also provoked confusion while selecting tomato types in the markets since less nutritional information of new products is revealed. Moreover, most studies usually considered specific nutrients such as amino acids, carotenoids, and polyphenols (e.g., flavonoids, flavanones, and flavones) of limited tomato cultivars and evaluated their functional effects. For example, a fully dried tomato contains 2–2.5% of free amino acids, mostly composed of glutamic acid, γ-aminobutyric acid, glutamine, and aspartic acid, which contribute to umami taste of the fruit (10, 11). Also, only carotenoids (lycopene and β-carotene) and polyphenols (apigenins and quercetins) were considered for comparison among tomato cultivars (12–14). However, global metabolite analysis of diverse tomato cultivars has not been done so far.

Recently, metabolomics approaches are harnessed to improve our fundamental understanding of metabolite composition in foods and plants. Most food metabolomics studies revealed a relation between metabolites and their bioactive functions, which can be potentially contributed to human health care (15–17). Intriguingly, plant metabolomics has provided important information about discriminant metabolites in plants and, concurrently, their correlations between metabolites and crop quality. Thus, metabolome information of plants could lead us to link specific metabolites with yield or quality relevant traits (e.g., colors, size, and nutrients) (18). However, there are scarce studies that considered the correlation between metabolome and bioactive functions of various tomato cultivars.

In order to understand global metabolic disparities in different cultivars of tomato, we chose five different types that represent various tomato cultivars. After that, we performed a non-targeted metabolomics analysis by using the gas chromatography time-of-flight mass spectrometry (GC-TOF-MS) and ultrahigh-performance liquid chromatography–linear trap quadrupole-orbitrap–tandem mass spectrometry (UHPLC-LTQ-Orbitrap-MS/MS) platforms. In addition, different metabolite contents of five tomato types were described in the metabolic pathway map. Finally, the correlation between metabolomics characteristics and other traits such as antioxidant activities and morphological traits was evaluated.

## Materials and Methods

### Chemicals and Reagents

Analytical-grade methanol, acetonitrile, and water were purchased from Fisher Scientific (Pittsburgh, PA, USA). The reagent-grade chemicals including 6-hydroxy-2,5,7,8-tetramethylchroman-2-carboxylic acid (trolox), hydrochloric acid, potassium persulfate, 2,2′-azinobis (3-ethylbenzothiazoline-6-sulfonic acid) diammonium salt (ABTS), hydrochloride, 2,4,6-tris(2-pyridyl)-trizine (TPTZ), iron(III) chloride hexahydrate, sodium acetate, acetic acid, sodium carbonate, sodium hydroxide, formic acid, methoxyamine hydrochloride, pyridine, and *N*-methyl-*N*-(trimethylsilyl)-trifluoroacetamide (MSTFA) were obtained from Sigma Chemical Co. (St. Louis, MO, USA).

### Sample Information and Preparation

Five types of tomato [chal tomato (*Lycopersicon esculentum* Mill.), tomato (*L. esculentum* Mill.), kumato (*Solanum lycopersicum* “Kumato”), cherry tomato (*L. esculentum* var. cerasiforme), and jujube-shaped cherry tomato (*L. esculentum* Mill.)] were purchased from 15 different local farms (Table 1). Each type of tomato cultivated in three different provinces were purchased from three distinct local markets. We rinsed tomato fruits with distilled water and wiped before being stored at −80°C. Each fruit was lyophilized for 4 days and then ground into a powder with a mortar and pestle. The powdered tomatoes were stored at −80°C until metabolite extraction.

**Table 1:** Information of five types of tomato samples and morphological characterization.

**No**.	**Types**	**Abbreviation**	**Harvest region^a^**	**Morphological characterization**		
				**Weight** **(g/fruit)^b^**	**Length** **(cm)**	**Width** **(cm)**
1	Chal tomato	CT	Gangwon-do	150.08 ± 10.12^a^	6.48 ± 0.29^a^	5.66 ± 0.18^b^
			Jeolla-do			
			Jeolla-do			
2	Tomato	ST	Jeolla-do	128.26 ± 12.40^b^	5.98 ± 0.39^b^	5.94 ± 0.54^a^
			Gangwon-do			
			Gangwon-do			
3	Kumato	KT	Gangwon-do	74.03 ± 7.30^c^	5.03 ± 0.17^c^	4.51 ± 0.29^c^
			Gangwon-do			
			Gangwon-do			
4	Cherry tomato	CH	Chungcheong-do	11.34 ± 1.32^d^	2.66 ± 0.14^d^	2.48 ± 0.14^e^
			Gangwon-do			
			Gangwon-do			
5	Jujube-shaped cherrytomato	JT	Chungcheong-do	10.97 ± 1.19^e^	2.33 ± 0.17^e^	3.78 ± 0.24^d^
			Jeolla-do			
			Jeolla-do			

a *Harvest regions of five tomato types are in the South Korea. Tomato cultivars were purchased from 15 different markets*.

b *Means of varietal group within columns separated by different letters are significantly distinguished according to the Duncan multiple range test at p-value < 0.05*.

### Sample Extraction

The powdered sample (100 mg) was extracted with 1 ml of 80% aqueous methanol using a MM 400 mixer mill (Retsch^®^ Haan, Germany) at a frequency of 30 s^−1^ for 10 min, followed by 5 min of sonication at 4°C (Hettich Zentrifugen Universal 320, Tuttlingen, Germany). Subsequently, the samples were centrifuged at 13,000 rpm for 10 min at 4°C, and the supernatants were filtered using 0.2-μm polytetrafluoroethylene syringe filters (Chromdisc, Daegu, Korea). The filtered supernatants were completely dried using a speed-vacuum concentrator (Biotron, Seoul, Korea). The dried samples were re-dissolved with 80% methanol to make a final concentration of 20,000 ppm for the following bioactivity assays and instrument analyses.

### Gas Chromatography Time-of-Flight Mass Spectrometry Analysis

For derivatization, 100 μl of the supernatant was taken in a fresh e-tube and completely dried. First, the oximation was performed by adding 50 μl of methoxyamine hydrochloride (20 mg/ml in pyridine) to the dried extract and incubated at 30°C for 90 min. Next, the silylation was performed by adding 50 μl of MSTFA to the reaction mixture, followed by a 37°C incubation for 30 min. The final concentration of the derivatized samples was set at 20,000 ppm; and daidzein (0.25 mg/ml) was used as the added internal standard (IS). All samples were filtered using Millex-GP 0.22-μm filters (Merck Millipore, Billerica, MA, USA) prior to the instrument analyses.

GC-TOF-MS analysis was performed using an Agilent 7890A GC system (Agilent Technologies, Palo Alto, CA, USA) coupled with an Agilent 7693 autosampler (Agilent Technologies) and a Pegasus HT TOF-MS (Leco Corporation, St. Joseph, MI, USA). The chromatographic separation was conducted by an Rtx-5MS column (30 m length × 0.25 mm inner diameter; J&W Scientific, USA) with a helium as carrier gas at a constant flow (1.5 ml/min). The analytical program for sample analysis was adopted from a previous study (19). We utilized three biological replicates for each type; and the analyses were performed in random order to reduce the bias and systematic errors.

### Ultrahigh-Performance Liquid Chromatography–Linear Trap Quadrupole-Orbitrap–Tandem Mass Spectrometry Analysis

The dried extracts were re-dissolved in 80% MeOH for UHPLC-LTQ-Orbitrap-MS/MS analysis. All analytes were set to 20,000 ppm by using daidzein (0.25 mg/ml) as IS.

A UHPLC system was equipped with a Vanquish binary pump H system (Thermo Fisher Scientific, Waltham, MA, USA) coupled with an autosampler and column compartment. Chromatographic separation was performed with a Phenomenex KINETEX^®^ C18 column (100 mm × 2.1 mm, 1.7 μm particle size; Torrance, CA, USA); and the injection volume was 5 μl. The column temperature was set at 40°C, and the flow rate was 0.3 ml/min. The MS data were collected in the range of 100–1,500 *m/z* (under both negative- and positive-ion modes) using an Orbitrap Velos Pro™ system, which was combined with an ion-trap mass spectrometer (Thermo Fisher Scientific) coupled with a HESI-II probe. All samples were analyzed based on the analytical methods described by Kwon et al. (20).

### Data Processing and Multivariate Statistical Analysis

The raw data sets of GC-TOF-MS were converted to the Net CDF format using the LECO Chroma TOF software (LECO Corporation). The UHPLC-LTQ-Orbitrap-MS/MS data were acquired with Xcalibur software (Thermo Fisher Scientific) and converted into Net CDF format. The CDF files were preprocessed with the MetAlign software package for alignment based on peak detection and retention time correction. For the multivariate statistical analysis, SIMCA-P+ software (Umetrics; Umeå, Sweden) was utilized.

The significantly different metabolites among five different tomato types were selected by variable importance projection (VIP > 0.7) values based on partial least squares-discriminant analysis (PLS-DA) score plot; and the significance test (*p*-value < 0.05) between experimental groups was tested by analysis of variance (ANOVA) and Duncan's multiple range tests using PASW Statistics 18 software (SPSS, Inc., Chicago, IL, USA). The selected metabolites were tentatively identified by comparing their retention time, mass fragment patterns, and elemental compositions and mass spectrum of analysis data with standard compounds under the same conditions in published papers and/or commercial databases such as the National Institute of Standards and Technology (NIST) Library (version 2.0, 2011, FairCom, Gaithersburg, MD, USA), The Dictionary of Natural Products (version 16:2, 2007, Chapman and Hall, USA), Wiley 8, BioCyc Database Collection (https://biocyc.org/), and the Human Metabolome Database (HMDB; http://www.hmdb.ca/). The correlation map analysis was obtained using PASW Statistics 18.0 software (SPSS Inc., Chicago, IL, USA) and constructed by MeV software (http://www.tm4.org/).

### Total Soluble Solids and Titratable Acidity

In order to measure the total soluble solids (TSSs) and titratable acidity (TA), fresh tomato fruits were squeezed using gauze to obtain the fresh juice of tomatoes. The TSS contents in 200 μl of fresh juice extract were measured using a portable refractometer for sugar measurements (Hanna Instruments, Inc., Padua, Italy). The TA was determined using the formal titration method, as described previously by Suh et al. (21). Each of the juice extracts (10 ml) was diluted with distilled water (40 ml), and the TA was estimated by titrating it using 0.1 N NaOH solution to pH 8.4. Taste index (TI) was derived from the TSS and TA values using the formula TI = TA + [SS/(20 * TA)] according to Figàs et al. (12).

### Determination of Antioxidant Activities by 2,2′-Azinobis (3-Ethylbenzothiazoline-6-Sulfonic Acid) Diammonium Salt and Ferric Reducing Antioxidant Power

Antioxidant activity tests including ABTS and ferric reducing antioxidant power (FRAP) were performed using the slightly modified procedures described elsewhere (22). For ABTS assay, 10 μl of the sample extract was added with 190 μl of the diluted ABTS solution (OD = 0.7 at 750 nm) in 96-well plates; and the mixture was incubated under dark condition for 7 min. The absorbance was measured at 750 nm using a microplate reader. For FRAP assay, 10 μl of each sample extract was added with 300 μl of FRAP reagent; and the reaction mixture was incubated for 6 min at 37°C in the 96-well microtiter plates. The absorbance was measured at 570 nm using a microplate reader. In all assays, the results were presented as the trolox equivalent antioxidant capacity (mM), ranging within 0.0078 and 1.000 mM using standard curve. We maintained three biological samples as well as analytical replicates for each assay.

### Determination of Total Phenolic and Flavonoid Contents

Total phenolic contents (TPCs) and total flavonoid contents (TFCs) were determined as described in previous study by Suh et al. (21). In order to evaluate the TPC, 20 μl of sample extracts was mixed with 100 μl of 0.2 N Folin–Ciocâlteu phenol reagent in a 96-well plate; and the mixture was incubated at room temperature in the dark. After incubating for 5 min, 80 μl of 7.5% NaCO_3_ was added to the mixture, which was then incubated for 6 min at room temperature. Finally, the absorbance was measured using a spectrophotometer at 750 nm. The results were expressed as gallic acid equivalents (ppm). For TFC measurement, 20 μl of sample extracts was added to 180 μl of 90% diethylene glycol and 20 μl of 1 N NaOH solution and then incubated for 60 min at room temperature in the dark. The absorbance was measured at 405 nm, and the results were presented as the naringin equivalents.

### Analysis of Carotenoids

Carotenoid analyses were performed according to previous study with a slight modification (23). Carotenoids were extracted from the tomato samples (25 mg) by adding 3 ml of ethanol containing 0.1% ascorbic acid (w/v). Mixtures vortexed for 20 s and placed in a water bath at 85°C for 5 min. The carotenoid extract was saponicated with potassium hydroxide (120 μl, 80%, w/w) at 85°C for 10 min. After saponification, samples were placed immediately on ice, and cold deionized water (1.5 ml) was added. β-Apo-80-carotenal (0.1 ml, 25 μg/ml) was used as an IS. Carotenoids were extracted twice with hexane (1.5 ml) by centrifugation at 1,200 × g to separate the layers. Aliquots of the extracts were dried and redissolved in 50:50 (v/v) dichloromethane/methanol before analysis. The content of carotenoid was analyzed by liquid chromatography–diode-array detection (LC-DAD) system, consisting of Shimadzu Nexera X2 LC-30AD Pump, Shimadzu SIL-30AC Autosampler and Shimadzu SPD-20A. Chromatographic separation was performed using a YMC carotenoid C30 column (250 mm × 4.6 mm × 5 μm particle size; YMC, Wilmington, NC); and the injection volume was 10 μl. The flow rate was 1 ml/min. The binary solvent system consisted of buffer A [methanol/water (92:8, v/v) with 10 mM of ammonium acetate] and buffer B (MTBE). The gradient parameters were set as follows: 20% solvent B was maintained initially for 1 min, followed by a linear increase to 100% solvent B over 19 min and then maintained for 1 min, with a gradual decrease to 20% solvent B over 1 min. The absorbance was measured at 450 nm. Identification of carotenoids was conducted by comparing retention times and absorbance spectra with those of standard chemicals.

## Results and Discussion

### Differences in Morphology, Physicochemical Characteristics, and Antioxidant Activities in Five Types of Tomato

**Figure 1:**
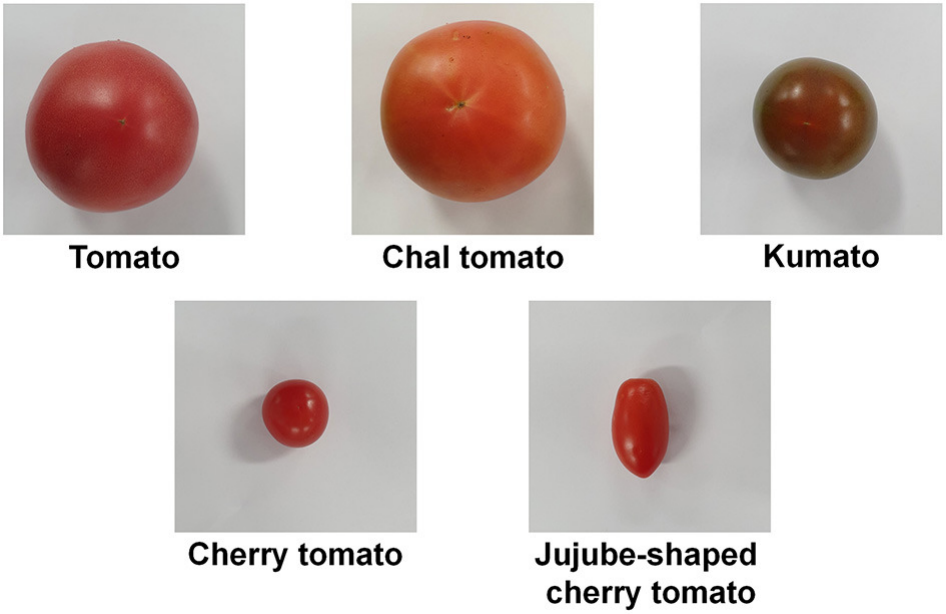
Photographs of five types of tomato.

**Figure 2:**
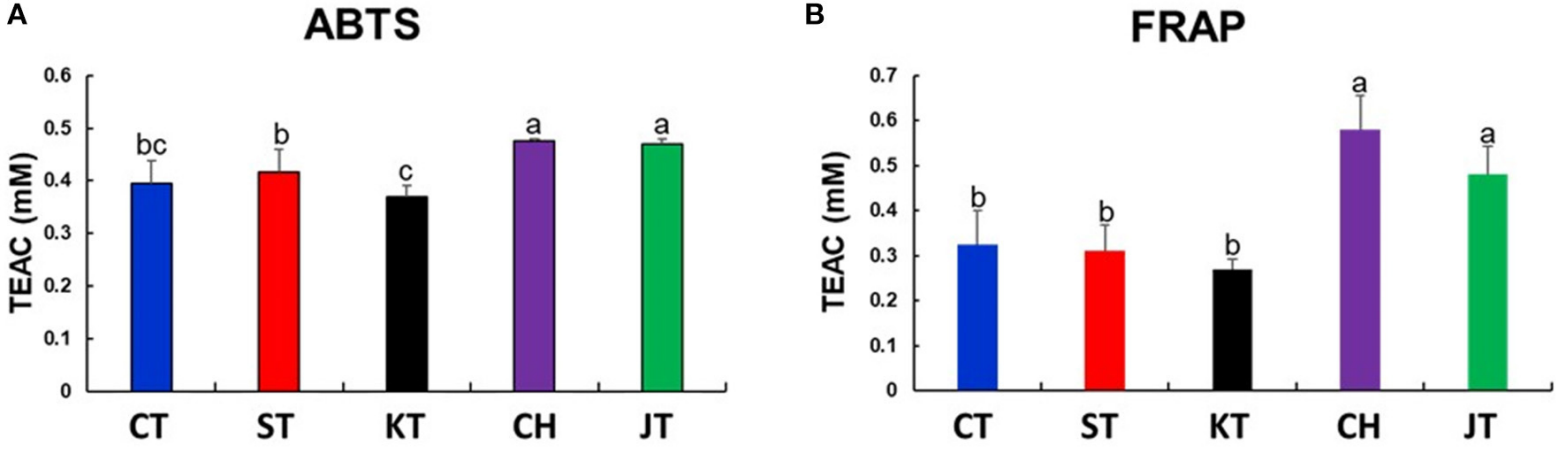
Results of antioxidant activities [2,2′-azinobis (3-ethylbenzothiazoline-6-sulfonic acid) diammonium salt (ABTS) **(A)** and ferric reducing antioxidant power (FRAP) **(B)**] in five types of tomato. Different letters in the bar graph indicate significant difference by ANOVA followed by Duncan's multiple range test (*p*-value < 0.05). TEAC, trolox equivalent antioxidant capacity; CT, chal tomato; ST, tomato; KT, kumato; CH, cherry tomato; JT, jujube-shaped cherry tomato.

We examined the morphological and physicochemical characteristics and antioxidant activities of five types of tomato to evaluate the palatability and quality parameters (Tables 1, 2 and Figures 1, 2). Figure 1 and Table 1 present photographs of five different tomatoes and their physical characteristics such as weight, length, and width. The morphological traits were varied as follows: the weight of fruit ranged from 10.97 (JT) to 150.08 g (CT); the length was within 2.33 (JT) and 6.48 cm (CT); the width of fruit was within 2.48 (CH) and 5.94 cm (ST). The evaluated morphologies of tomatoes were varied depending on the fruit types. These data indicate various morphological traits of each cultivar.

**Table 2:** Physicochemical characters of five types of tomato samples.

**Tomato types**	**Chal tomato** **(CT)**	**Tomato** **(ST)**	**Kumato** **(KT)**	**Cherry tomato** **(CH)**	**Jujube-shaped** **cherry tomato** **(JT)**
**Quality parameters**					
Total soluble solids (Brix)	5.36 ± 0.45^b^	4.30 ± 0.36^c^	5.29 ± 0.21^b^	6.16 ± 0.32^b^	7.64 ± 2.07^a^
Titratable acidity (% acid)	0.40 ± 0.04^a^	0.23 ± 0.03^d^	0.34 ± 0.01^c^	0.39 ± 0.06^ab^	0.36 ± 0.04^bc^
Taste index	1.16 ± 0.07^bc^	1.07 ± 0.04^c^	1.12 ± 0.03^bc^	1.19 ± 0.03^b^	1.41 ± 0.20^a^
Total phenolic content (mg GEC/g extract)	47.14 ± 11.43^c^	81.96 ± 20.90^b^	33.57 ± 6.98^c^	139.76 ± 18.02^a^	127.46 ± 20.91^a^
Total flavonoid content (mg NEC/g extract)	0.75 ± 0.14^c^	1.26 ± 0.28^b^	1.13 ± 0.20^bc^	2.66 ± 0.30^a^	2.71 ± 0.77^a^
**Carotenoids (mg/100 g DW)**					
Lutein	1.57 ± 0.02^c^	0.99 ± 0.01^e^	2.24 ± 0.01^a^	1.25 ± 0.01^d^	1.67 ± 0.01^b^
α-Carotene	0.43 ± 0.00^d^	0.41 ± 0.02^d^	0.52 ± 0.01^c^	0.63 ± 0.05^b^	0.81 ± 0.01^a^
β-Carotene	6.82 ± 0.17^c^	6.57 ± 0.07^c^	9.07 ± 0.04^b^	9.00 ± 0.28^b^	10.50 ± 0.16^a^
Lycopene	34.58 ± 0.65^d^	51.32 ± 0.71^c^	25.78 ± 1.03^e^	53.62 ± 0.48^b^	77.27 ± 0.63^a^

*Means of varietal group within columns separated by different letters are distinguished according to the Duncan multiple range test at p-value < 0.05*.

The physicochemical characteristics, TSS and TA, were evaluated (Table 2). The TSS average of JT and ST was the highest and lowest, respectively. The TSS values of CH, CT, and KT were insignificantly different. TA indicates the relative acidity and sourness of fruits. Among the five types, the highest TA was observed in CT, while the lowest in ST. JT showed the highest TI, representing flavor intensity of fruits, while the lowest value was observed with ST.

Tomatoes are a good source of phenolic compounds and carotenoids, but the compositional variation of these compounds in various types has not been well-documented. Thus, we also determined the TPC, TFC, carotenoid contents, and antioxidant activities (ABTS and FRAP) (Table 2 and Figures 2A,B). TPC and TFC levels of CH and JT were higher than those of other types (Table 2). Also, CH and JT showed higher antioxidant activities (indicated by ABTS and FRAP assays) than did other tomatoes. Surprisingly, the antioxidant activity showed a similar tendency with TPC. Next, we measured carotenoid contents since they are generally known as bioactive compounds and related to colors of fruits. JT presented the highest level of lycopene (77.27 mg/100 g DW) while KT, where the color of the fruit varies from reddish brown to purple, was the lowest (25.78 mg/100 g DW) among all tomatoes. α-Carotene and β-carotene were the highest in JT, whereas lutein was the highest in KT. Notably, JT showed higher levels of carotenoid contents, TPC, antioxidant activities, and TI. As a result, statistically significant differences were observed in the above-mentioned parameters among five types of tomato.

### Non-targeted Metabolite Profiling of Five Types of Tomato

**Figure 3:**
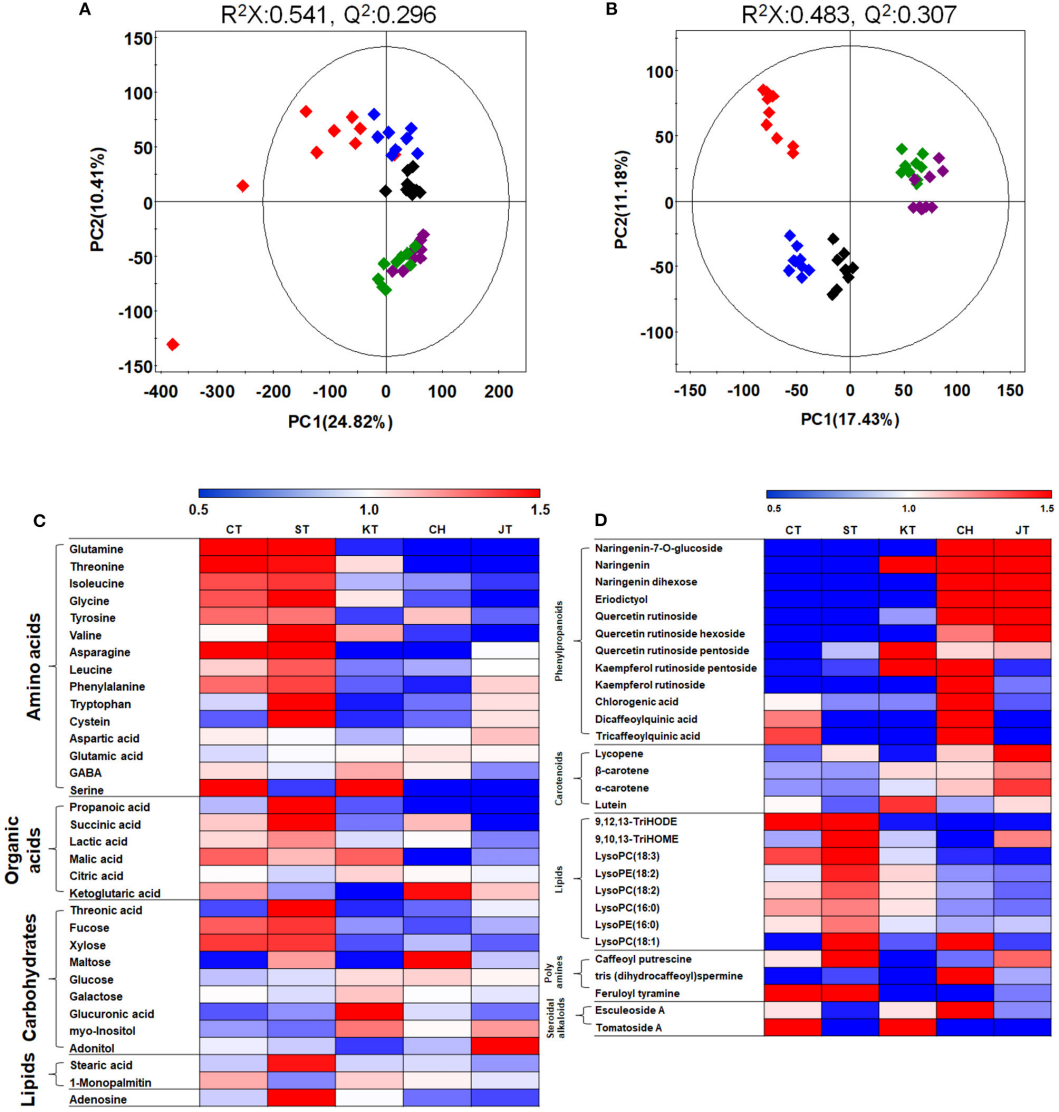
Principal component analysis (PCA) **(A,B)** score plots for metabolites in five types of tomato based on GC-TOF-MS **(A)** and UHPLC-LTQ-Orbitrap-MS/MS **(B)** data set. Heat map analysis of five tomato types based on GC-TOF-MS **(C)** and UHPLC-LTQ-Orbitrap-ESI-MS/MS **(D)** data. Heat map representation of the relative abundance of significantly discriminant metabolites (VIP > 0.7, *p*-value < 0.05) based on PLS-DA model (Supplementary Figure 1). Chal tomato (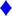), tomato (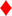), kumato (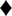), cherry tomato (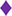), and jujube-shaped cherry tomato (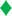) samples. GC-TOF-MS, gas chromatography time-of-flight mass spectrometry; UHPLC-LTQ-Orbitrap-MS/MS, ultrahigh-performance liquid chromatography–linear trap quadrupole-orbitrap–tandem mass spectrometry; VIP, variable importance projection; PLS-DA, partial least squares-discriminant analysis.

In order to analyze global metabolites in five tomato types, metabolite profiling was performed by GC-TOF-MS and UHPLC-LTQ-Orbitrap-MS/MS. After that, to evaluate differences of five tomatoes based on metabolites, we performed the principal component analysis (PCA) score plot. It revealed that five types of tomato are distinguished from each other and clustered depending on their types (Figures 3A,B). The PLS-DA score plot showed similar results (Supplementary Figures 1A,B). In order to investigate distinctive metabolites among types, we selected significantly discriminated metabolites based on the VIP value (>0.7) using PLS-DA (Supplementary Figure 1) and *p*-value (<0.05). The differences among tomato samples were observed mostly depending on their types, based on the PCA score plot, even though tomato samples were collected from 15 different markets (Figures 3A,B). Interestingly, common (CT and ST) and cherry (CH and JT) tomato cultivars were significantly distinguished based on our PCA, concurrently with the physicochemical and functional properties.

A total of 58 distinguished metabolites were identified, of which 32 and 26 metabolites were identified by GC-TOF-MS and UHPLC-LTQ-Orbitrap-MS/MS, respectively (Supplementary Tables 1, 2). These identified metabolites include 15 amino acids, seven organic acids, eight carbohydrates, 12 phenylpropanoids (two naringenin derivatives, eriodictyol, three quercetin derivatives, two kaempferol derivatives, and three quinic acid derivatives), 10 lipids (two fatty acids, six lysophospholipids, two oxylipins), three polyamines, and two glycoalkaloids. Surprisingly, the common tomatoes, distinguished from cherry tomatoes in PCA, showed higher contents of primary metabolites such as amino acids, organic acids, and lipids, than did cultivars of cherry tomato, concurrently with the size and weight (Figure 3C and Table 1). For visualization of significantly different metabolites, all metabolites were depicted in a heat map (Figures 3C,D). Based on that, most amino acids, organic acids, and lipids were observed higher in CT and ST than other tomatoes. Particularly, the levels of phenylpropanoid such as eriodictyol, naringenins, and quercetins were significantly higher in CH and JT. In CH, the levels of kaempferol derivatives and quinic acids were higher than those of other tomatoes.

### Comparison of Metabolic Pathway and Correlation Between Antioxidant Activities, Morphological Traits, and Discriminant Metabolites in Five Types of Tomato

**Figure 4:**
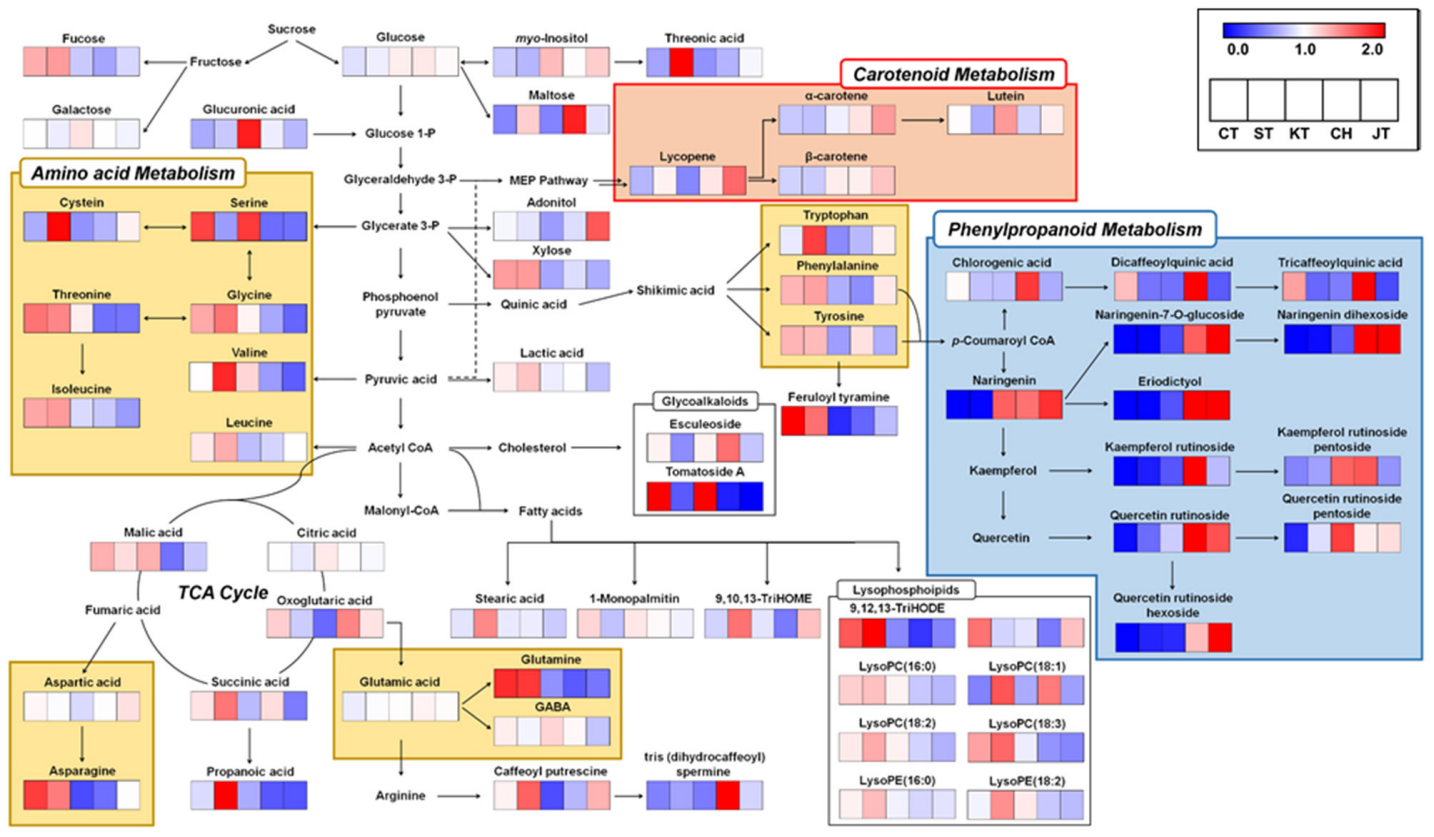
Schematic diagram of the metabolic pathway and relative levels of metabolites in five types of tomato. The relative levels are shown as fold-changes normalized using the average of all values. The metabolic pathway was modified based on the KEGG database (http://www.genome.jp/kegg/). C, chal tomato; T, tomato; K, kumato; CH, cherry tomato; JT, jujube-shaped cherry tomato.

We constructed biosynthetic pathways of tomatoes based on the identified metabolites (Figure 4). Also, relative levels of each metabolite are represented in the metabolic pathway map. CT and ST contain higher levels of primary metabolite such as amino acids (yellow box in Figure 4), organic acids, and lipids. For instance, glycerate 3-phosphate-derived amino acids (serine, glycine, threonine, and isoleucine), shikimate-derived metabolites (tryptophan, phenylalanine, and tyrosine) and tricarboxylic acid (TCA) cycle-derived amino acids (asparagine and glutamine) were higher in CT and ST. Generally, amino acids in fruits play an important role in organoleptic qualities by enhancing taste and flavor of fruits (24, 25). Interestingly, in our data, glutamic acid, γ-aminobutyric acid, and aspartic acid, which are also TCA cycle-derived amino acids relevant to umami taste, were observed to be of similar abundance among the five types (26), although we observed that the similar levels of umami taste-relevant amino acids and TI values, determined by TSS and TA values, are represented distinctly in different types of tomato (Table 2). Because the tastes and flavors are not only determined by sweetness and sourness, the taste of various tomatoes might be relevant with total composition of sugars, organic acids, and free amino acids (27). In addition, malic acid, influencing sourness of fruit, was abundantly observed in common tomatoes (28). Similarly, lipids, including stearic acid, 1-monopalmitin, and 9,12,13-TriHODE and several lysophospholipids such as LysoPC(16:0), LysoPC(18:2), LysoPE(16:0), and LysoPC(18:3), derived from acetyl-CoA and malonyl-CoA, were more abundant in CT and ST. In summary, we observed higher abundance of amino acids, organic acids, and lipids compounds in the ST and CT. These metabolites were significantly discriminant in the other tomatoes and correlated positively with size and weight. It is noted that at a higher level of secondary metabolites, likewise phenylpropanoids and carotenoids were observed in CH and JT (blue and red boxes in Figure 4). Few metabolites synthesized from chlorogenic acid showed a higher level in CT and CH. Unlike the metabolites synthesized from chlorogenic acid, other phenylpropanoids were relatively abundant in CH and JT. Generally, tomatoes contain quercetin, naringenin, and chlorogenic acid as the main phenolic compounds (29). These phenolic compounds, because of their structure, are very efficient scavengers of peroxyl radicals (30). Notably, several carotenoids, synthesizing via MEP pathway, were observed to be higher in JT. Lutein was higher in KT than other cultivars of tomato, whereas lycopene and α-carotene, the precursors of lutein, were lower. It seems that KT has the ability to synthesize more lutein than other tomatoes. Lycopene, which is one of the most well-known antioxidant compounds in tomatoes, was detected in all tomatoes (31). Especially, higher contents of lycopene were observed in CH and JT. Another carotenoid of bioactive relevance present in the tomato fruit is β-carotene, although its levels are normally much lower than those of lycopene (14). It was reported that intake of lycopene and β-carotene is correlated with reduction of cancer and cardiovascular disease risks (32, 33).

**Figure 5:**
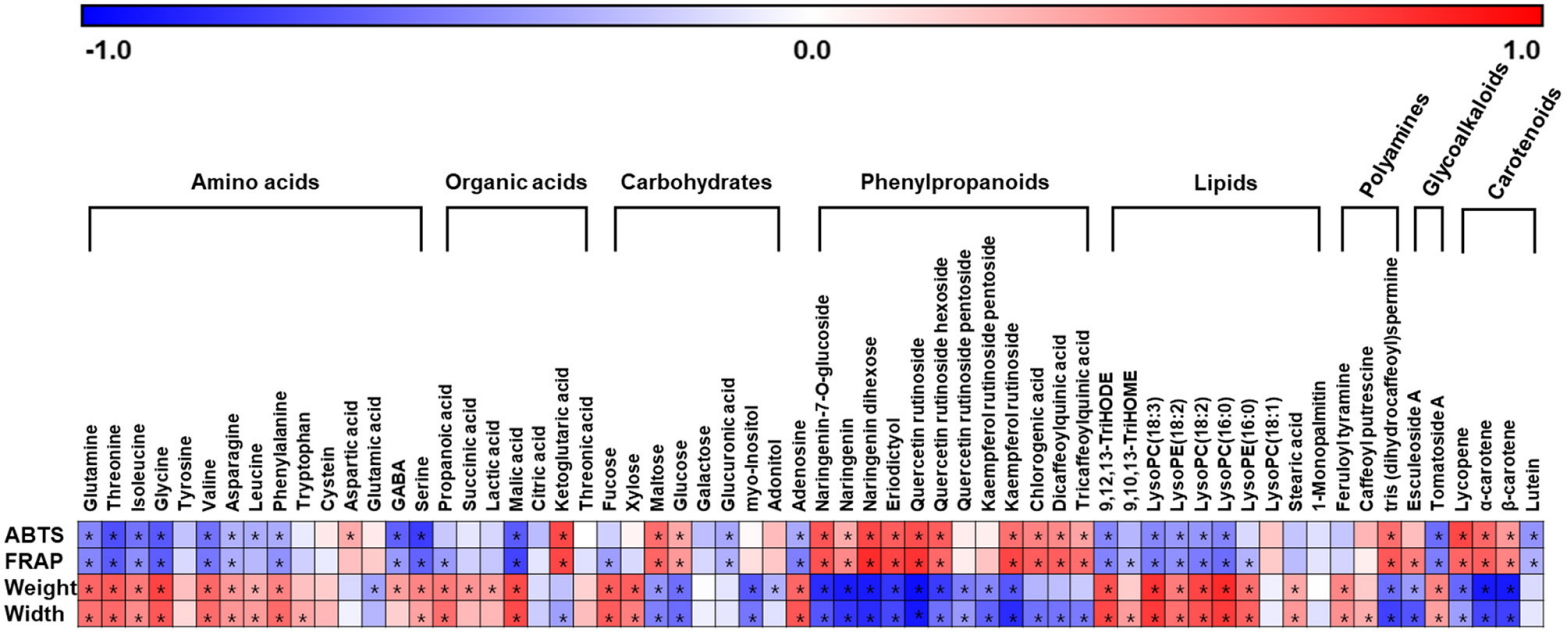
Correlation map between the metabolite levels and observed ABTS, FRAP, weight, and width in five types of tomato. Each metabolite is identified as significantly different metabolites through PLS-DA model (Supplementary Figure 1). Each square implies Pearson's correlation coefficient between metabolites and assayed activities. The red color indicates a positive (0 < *r* < 1) correlation, and the blue colors indicate a negative (−1 < *r* < 0) correlation. Asterisks indicate *p-*values < 0.05. ABTS, 2,2′-azinobis (3-ethylbenzothiazoline-6-sulfonic acid) diammonium salt; FRAP, ferric reducing antioxidant power; PLS-DA, partial least squares-discriminant analysis.

To evaluate the relation between metabolites and traits of tomatoes, we performed a correlation analysis using discriminant metabolites, phenotype, and antioxidant activities of tomatoes (Figure 5). As a result, phenylpropanoids (quercetins, naringenins, and quinic acids), mainly existing in both CH and JT, showed a positive correlation with antioxidant activities. This indicates that the relative abundance of phenylpropanoids is closely related to high antioxidant activities. On the other hand, several amino acids, organic acids, lipids, and carbohydrates (e.g., fucose and xylose) were more abundant in CT and ST, showing a positive correlation with phenotype (weight, length, and width). In summary, through non-targeted metabolite analysis, we detected 58 significantly different metabolites in five types of tomato including amino acids, lipids, carotenoids, and phenylpropanoids. Based on the metabolite profiling, antioxidant activities, and morphological traits, we could explain the correlation between phenotypes and metabolite levels in various tomatoes.

## Conclusion

Assorted tomatoes have been developed to attract more consumers. Thus, we can guess that various tomatoes with different appearances may contain different nutrients and have different effects on human (e.g., bioactivities). Therefore, in this study, we performed non-targeted metabolite profiling of five representative tomato types to evaluate the metabolite differences. Subsequently, the metabolic pathway map of tomatoes was constructed to infer metabolic differences in each type. Quality (TA, TSS, size, and weight) and functional properties (TPC, TFC, ABTS, and FRAP) were also determined to evaluate overall commercial values of representative tomatoes. As a result, we observed the higher levels of TSS, TI, TPC, TFC, and antioxidant activity and secondary metabolites in cherry tomatoes. The higher level of secondary metabolites might be associated with its high antioxidant activities. On the other hand, common tomatoes (CT and ST) showed higher levels of primary metabolites and morphological traits, which can enhance flavor and taste of fruits. Our study of comparing functional properties among the five tomato types can provide useful information to improve tomato cultivars. Moreover, based on our metabolite profiling and functional studies, we suggest consumers to choose different tomato types to take various nutrients. In the future, in order to deeply and comprehensively understand the metabolite disparity of tomato types, multi-omics data such as genome and transcriptome of different types should be integrated with metabolome data.

## Data Availability Statement

The original contributions presented in the study are included in the article/Supplementary Material, further inquiries can be directed to the corresponding author/s.

## Author Contributions

HM performed data processing and statistical analysis and drafted the manuscript. MK performed measurement of the physicochemical characters. MK and DS performed metabolite profiling. N-RL and SS revised the manuscript. CL supervised and took complete responsibility for this project. All authors contributed to the article and approved the submitted version.

## Conflict of Interest

The authors declare that the research was conducted in the absence of any commercial or financial relationships that could be construed as a potential conflict of interest.
