# Supplementary material for: Comparing Metabolites and Functional Properties of Various Tomatoes Using Mass Spectrometry-Based Metabolomics Approach
Source: Front Nutr. 2021 Apr 8;8:659646. doi: 10.3389/fnut.2021.659646 (PMC8060453; doi:10.3389/fnut.2021.659646)
Supplement: Supplementary file 1 [file Table_1.DOCX]

Supplementary Material

**Supplementary Table 1.** Primary metabolites identified by GC-TOF-MS in 5 varieties of tomato.

| **No.** | **Tentative  identification** | **GC-TOF-MS** | | | | |  |
| --- | --- | --- | --- | --- | --- | --- | --- |
|  |  | **RT**  **(min) ^a^** | **Mass** | **Mass Fragment** | **TMS ^b^** | **ID ^c^** |  |
|  |  |  |  |  |  |  |  |
| *Amino acids* | | | | |  |  |  |
| 1 | Leucine | 5.88 | 86 | 86 61 103 70 59 57 188 | 1 | STD |  |
| 2 | Isoleucine | 6.11 | 86 | 86 69 56 146 103 130 61 | 1 | STD |  |
| 3 | Valine | 6.51 | 218 | 218 59 74 145 100 219 | 2 | STD |  |
| 4 | Glycine | 7.37 | 174 | 174 86 100 59 175 133 | 3 | STD |  |
| 5 | Serine | 7.88 | 204 | 204 100 218 188 133 205 | 3 | STD |  |
| 6 | Threonine | 8.125 | 117 | 117 57 101 219 218 100 | 3 | STD |  |
| 7 | Aspartic acid | 9.27 | 232 | 100 232 117 75 233 133 | 3 | STD |  |
| 8 | GABA | 9.34 | 174 | 174 216 246 117 100 86 | 3 | STD |  |
| 9 | Cysteine | 9.56 | 220 | 220 100 218 221 115 57 | 3 | STD |  |
| 10 | Glutamic acid | 10.04 | 128 | 128 246 84 156 100 56 | 3 | STD |  |
| 11 | Phenylalanine | 10.15 | 192 | 218 192 100 91 219 59 | 2 | STD |  |
| 12 | Asparagine | 10.47 | 116 | 116 132 231 100 188 141 | 3 | STD |  |
| 13 | Glutamine | 11.218 | 156 | 156 155 245 131 128 100 | 3 | STD |  |
| 14 | Tyrosine | 12.442 | 354 | 354 218 100 219 179 220 | 3 | STD |  |
| 15 | Tryptophan | 14.16 | 202 | 202 203 74 103 291 132 | 3 | STD |  |
| *Organic acids* | |  |  |  |  |  |  |
| 16 | Lactic acid* | 4.9 | 117 | 117 191 66 148 59 | 2 | STD |  |
| 17 | Succinic acid | 7.43 | 247 | 55 247 149 61 172 | 2 | STD |  |
| 18 | Propanoic acid | 7.6 | 103 | 189 133 102 117 292 | 3 | STD |  |
| 19 | Malic acid | 9 | 335 | 55 233 245 101 189 117 | 3 | STD |  |
| 20 | Citric acid | 11.6 | 273 | 273 274 67 59 69 466 | 4 | STD |  |
| 21 | Ketoglutaric acid | 11.82 | 173 | 173 55 157 147 129 316 | 2 | STD |  |
| *Carbohydrates* | |  |  |  |  |  |  |
| 22 | Threonic acid | 9.61 | 292 | 117 292 103 102 130 220 | 4 | STD |  |
| 23 | Xylose | 10.397 | 307 | 103 217 147 307 133 | 4 | STD |  |
| 24 | Fucose | 10.942 | 117 | 277 219 201 117 133 | 4 | STD |  |
| 25 | Adonitol | 11.043 | 217 | 217 205 75 59 133 148 | 5 | MS |  |
| 26 | Galactose | 12.312 | 324 | 160 205 103 117 206 320 | 5 | STD |  |
| 27 | Glucose | 12.372 | 160 | 217 205 160 157 133 229 | 5 | STD |  |
| 28 | Glucuronic acid | 12.97 | 333 | 333 160 334 171 189 161 | 3 | STD |  |
| 29 | *myo-*Inositol | 13.558 | 191 | 191 217 318 129 133 148 | 6 | STD |  |
| 30 | Maltose | 17.81 | 204 | 204 103 129 217 361 160 | 8 | STD |  |
| *Fatty acids* | |  |  |  |  |  |  |
| 31 | Stearic acid | 14.13 | 117 | 117 129 132 55 145 57 | 1 | STD |  |
| 32 | 1-Monopalmitin | 16.02 | 371 | 57 129 103 371 117 203 | 2 | STD |  |
| *Others* | |  |  |  |  |  |  |
| 33 | Adenosine | 16.37 | 236 | 230 236 103 245 192 217 | 4 | STD |  |

* Differential metabolites were selected based on the VIP value (>0.7) and *p*-value (<0.05) from the PLS-DA model in Figure S1A. ^a^ Retention time; ^b^ Trimethylsilyl; ^c^ Identification. MS, mass spectrum compared with the National Institute of Standards and Technology (NIST) database and in-house libraries; STD, mass spectrum consistent with that of the standard compounds.
