# Supplementary material for: Comparing Metabolites and Functional Properties of Various Tomatoes Using Mass Spectrometry-Based Metabolomics Approach
Source: Front Nutr. 2021 Apr 8;8:659646. doi: 10.3389/fnut.2021.659646 (PMC8060453; doi:10.3389/fnut.2021.659646)
Supplement: Supplementary file 2 [file Table_2.DOCX]

Supplementary Material

**Supplementary Table 2.** Differential secondary metabolites identified by UHPLC-LTQ-Orbitrap-MS/MS in 5 varieties of tomato.

| **No.** | **RT^a^** | **Tentative  Metabolite^b^** | **UHPLC-LTQ-Orbitrap-MS/MS** | | | | | | **References** |
| --- | --- | --- | --- | --- | --- | --- | --- | --- | --- |
|  | **(min)** |  | **[M-H]^-^** | **[M+H]^+^** | **M.W.^c^** | **M.F.^d^** | **Delta ppm** | **MS^n^ fragments** |  |
| ***Phenylpropanoids*** | | | | | | | | | |
| 1 | 3.86 | Chlorogenic acid | 353.0903 | 355.102 | 354 | C16H17O9 | 7.15 | 191, 179, 173 | Ref[S1] |
| 2 | 5.33 | Dicaffeoylquinic acid | 515.122 | 517.1241 | 516 | C25H23O12 | 4.874 | 353, 317, 299, 173 | Ref[S2] |
| 3 | 5.91 | Tricaffeoylquinic acid | 677.1529 | 679.2969 | 678 | C34H29O15 | 4.677 | 515, 353, 299 | Ref[S3] |
| 4 | 5.48 | Naringenin-*O*-glucoside | 433.1172 | 435.1276 | 434 | C21H21O10 | 5.472 | 313, 295, 271, 253 | Ref[S4] |
| 5 | 3.86 | Naringenin dihexose | 595.1691 | 597.1808 | 596 | C27H31O15 | 5.472 | 385, 355, 271, 235 | Ref[S4] |
| 6 | 6.33 | Naringenin | 271.0622 | 273.0753 | 272 | C15H11O5 | 2.89 | 253, 177, 151 | Ref[S4] |
| 7 | 5.89 | Eriodictyol | 287.0582 | 289.07 | 288 | C15H11O6 | 7.694 | 269 151, 135 | Ref[S4] |
| 8 | 4.79 | Quercetin rutinoside | 609.1478 | 611.1613 | 610 | C27H29O16 | 2.827 | 609, 301, 300, 271, 255 | Ref[S4] |
| 9 | 4.59 | Quercetin rutinoside pentoside | 741.1912 | 743.2017 | 742 | C32H37O20 | 3.769 | 741, 609, 300, 271, 255 | Ref[S3] |
| 10 | 3.98 | Quercetin rutinoside hexoside | 771.2007 | 773.2121 | 772 | C33H41O21 [pos] | -1.79 | 771, 753, 609, 301 | Ref[S4] |
| 11 | 5.01 | Kaempferol rutinoside | 593.1533 | 595.4026 | 594 | C27H29O15 | 3.585 | 593, 285, 267, 257, 241 | Ref[S3] |
| 12 | 4.77 | Kaempferol rutinoside pentoside | 725.1949 | 727.2072 | 726 | C32H37O19 | 1.983 | 725, 593, 575, 357, 327, 285 | Ref[S3] |
| ***Lipids*** | | | | | | | | | |
| 13 | 6.37 | 9,12,13-TriHODE | 327.2202 | 351.2137^e^ | 328 | C18H31O5 | -0.637 | 309, 291, 283, 229, 171 | Ref[S5] |
| 14 | 6.62 | 9,10,13-TriHOME | 329.2351 | 353.2293^e^ | 330 | C26H21O10 | -1.811 | 329, 311, 293, 201 | Ref[S5] |
| 15 | 8.12 | LysoPC(18:3) | 562.3171 ^f^ | 518.3221 | 517 | C26H49NPO7 [pos] | -3.928 | 562, 502, 277 | Ref[S6] |
| 16 | 8.43 | LysoPE(18:2) | 476.2824 | 478.2929 | 477 | C23H45NPO7 [pos] | -3.002 | 478, 460, 337, 198 [pos] | Ref[S6] |
| 17 | 8.52 | LysoPC(18:2) | 564.3337 ^f^ | 520.3399 | 519 | C26H51NPO7 [pos] | -3.009 | 520, 502, 184 [pos] | Ref[S6] |
| 18 | 8.81 | LysoPC(16:0) | 540.3334 ^f^ | 496.3381 | 495 | C24H51NPO7 [pos] | -3.356 | 496, 478, 419, 184 [pos] | Ref[S6] |
| 19 | 9.02 | LysoPC(18:1) | 566.3492 ^f^ | 522.3534 | 521 | C26H53NPO7 | -3.878 | 522 504, 445 [pos] | Ref[S6] |
| 20 | 8.71 | LysoPE(16:0) | 452.2804 | 454.2913 | 453 | C21H43NPO7 | 4.638 | 391, 365, 301, 255 | Ref[S6] |
| ***Steroidal alkaloids*** | | | | | | | | | |
| 21 | 4.81 | Esculeoside A | 1268.592 | 1270.605 | 1269 | C58H94NO29 | 0.08 | 1269, 1037, 1108, 975, 932, 914, 752, 590 | Ref[S7] |
| 22 | 5.41 | Tomatoside A | 1081.544 | 1105.535^e^ | 1082 | C51H85O24 | 0.521 | 1081, 920, 758, 597 | Ref[S8] |
| ***Polyamines*** | | | | | | | | | |
| 23 | 1.77 | Caffeoyl putresine | 249.1264 | 251.1387 | 250 | C13H19N2O3 | -1.19 | 249, 207, 135 | Ref[S9] |
| 24 | 4.69 | tris (dihydrocaffeoyl) spermine | 693.3532 | 695.3636 | 694 | C37H49N4O9 | 3.876 | 693, 571, 529, 407, 365 | Ref[S9] |
| 25 | 5.82 | Feruloyl tyramine | 312.1265 | 314.1372 | 313 | C18H18NO4 | 5.052 | 312, 177, 144, 117 | Ref[S10] |

**Supplementary references**

1. Ricci A, Cirlini M, Calani L, Bernini V, Neviani E, Del Rio D, et al. In vitro metabolism of elderberry juice polyphenols by lactic acid bacteria. *Food Chem* (2019) 276; 692-699. doi: 10.1016/j.foodchem.2018.10.046
2. Zhong RF, Xu GB, Wang Z, Wang AM, Guan HY, Li J, et al. Identification of anti-inflammatory constituents from Kalimeris indica with UHPLC-ESI-Q-TOF-MS/MS and GC–MS. *J Ethnopharmacol* (2015) 165; 39-45. doi: 10.1016/j.jep.2015.02.034
3. Anton D, Bender I, Kaart T, Roasto M, Heinonen M, Luik A, et al. Changes in polyphenols contents and antioxidant capacities of organically and conventionally cultivated tomato (*Solanum lycopersicum* L.) fruits during ripening. *Int J Anal Chem* (2017) 2017. doi: 10.1155/2017/2367453
4. Vallverdú-Queralt A, Jáuregui O, Di Lecce G, Andrés-Lacueva C, Lamuela-Raventós RM. Screening of the polyphenol content of tomato-based products through accurate-mass spectrometry (HPLC–ESI-QTOF). *Food chem* (2011) 129(3); 877-883. doi: 10.1016/j.foodchem.2011.05.038
5. Son SY, Lee S, Singh D, Lee NR, Lee, DY, Lee CH. Comprehensive secondary metabolite profiling toward delineating the solid and submerged-state fermentation of *Aspergillus oryzae* KCCM 12698. *Front Microbiol* (2018) 9; 1-12. doi: 10.3389/fmicb.2018.01076
6. Wei Z, Xi J, Gao S, You X, Li N, Cao Y, et al. Metabolomics coupled with pathway analysis characterizes metabolic changes in response to BDE-3 induced reproductive toxicity in mice*. Sci Rep* (2018) 8(1); 5423. doi: 10.1038/s41598-018-23484-2
7. Iijiman Y, Fujiwara Y, Tokita T, Ikeda T, Nohara T, Aoki K, Shibata D. Involvement of ethylene in the accumulation of esculeoside A during fruit ripening of tomato (*Solanum lycopersicum*). *J Agric Food Chem* (2009) 57(8); 3247-3252. doi: 10.1021/jf8037902
8. Gómez-Romero M, Segura-Carretero A, Fernández-Gutiérrez A. Metabolite profiling and quantification of phenolic compounds in methanol extracts of tomato fruit. *Phytochemistry* (2010) 71(16); 1848-1864. doi: 10.1016/j.phytochem.2010.08.002
9. Narváez-Cuenca CE, Vincken JP, Gruppen H. Identification and quantification of (dihydro) hydroxycinnamic acids and their conjugates in potato by UHPLC–DAD–ESI-MS^n^. *Food Chem* (2012) 130(3); 730-738. doi: 10.1016/j.foodchem.2011.04.050
10. Voynikov Y, Zheleva-Dimitrova D, Gevrenova R, Lozanov V, Zaharieva MM, Tsvetkova I, et al. Hydroxycinnamic acid amide profile of *Solanum schimperianum* Hochst by UPLC-HRMS. *Int J Mass Spectrom* (2016) 408; 42-50. doi: 10.1016/j.ijms.2016.08.008
