# Supplementary figures and images for: Comparing Metabolites and Functional Properties of Various Tomatoes Using Mass Spectrometry-Based Metabolomics Approach
Source: Front Nutr. 2021 Apr 8;8:659646. doi: 10.3389/fnut.2021.659646 (PMC8060453; doi:10.3389/fnut.2021.659646)

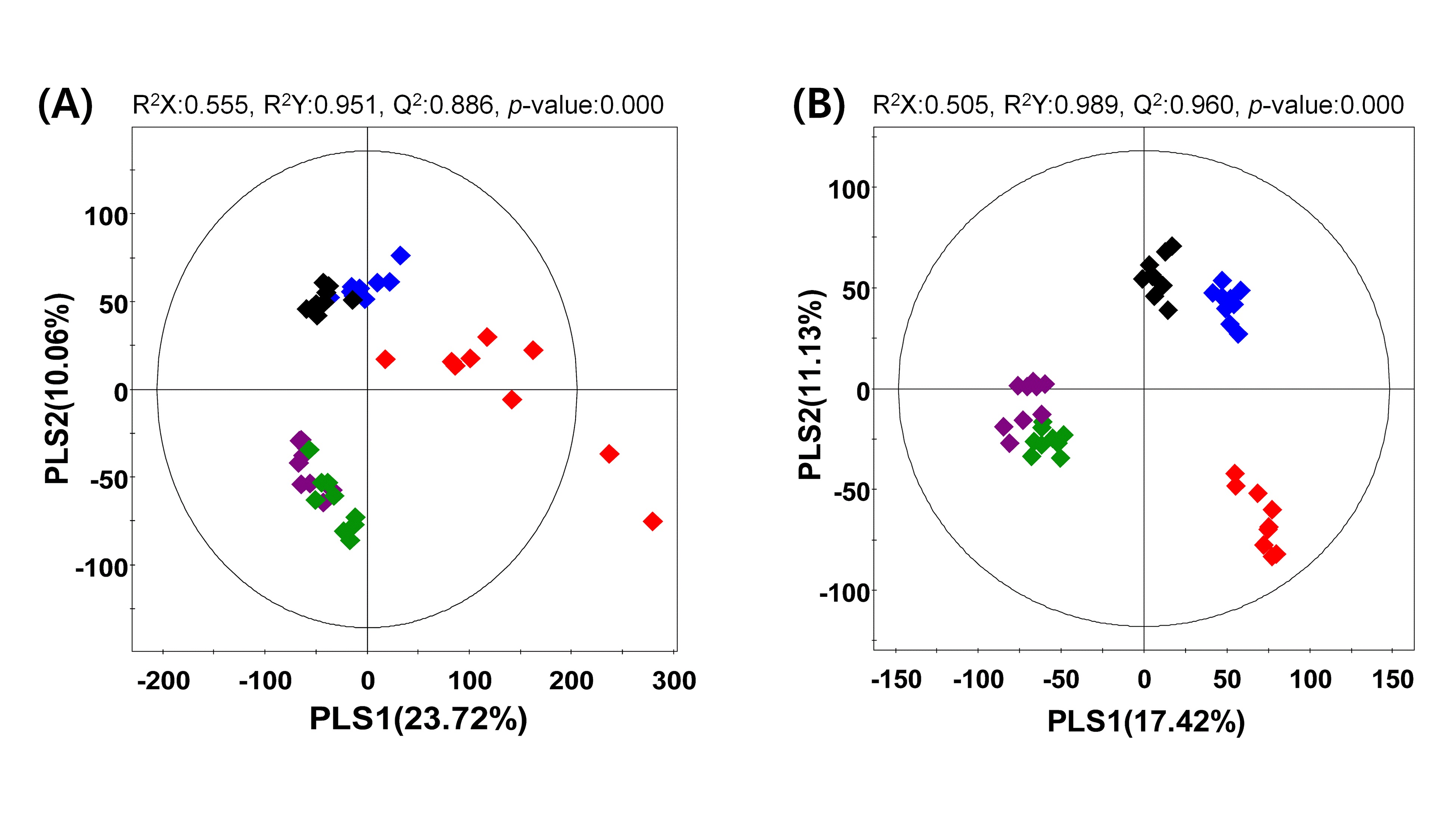

Supplement: Supplementary file 3 [file Image_1.jpg]
